# Prevalence of excess body weight and associated factors among secondary school adolescent girls in northern Tanzania: a cross-sectional study

**DOI:** 10.11604/pamj.2020.37.253.25349

**Published:** 2020-11-19

**Authors:** Anna Tengia-Kessy, Jackline Narcis Killenga

**Affiliations:** 1 Muhimbili University of Health and Allied Sciences, Dar es Salaam, Tanzania,; 2 Same District Council, Kilimanjaro, Tanzania

**Keywords:** Overweight, adolescents, dietary habits, healthy eating, Tanzania

## Abstract

**Introduction:**

excess body weight among adolescents is on the increase and has become a global public health challenge. It is likely to persist to adulthood, exposing to risk of developing chronic diseases. However, there is insufficient information on the prevalence of excess body weight and associated factors among adolescent girls in secondary schools in northern Tanzania.

**Methods:**

this cross-sectional study involved 400 secondary school adolescent girls, selected by multi-stage cluster sampling. A self-administered questionnaire was used to gather information. Anthropometric measurements were taken and body mass index calculated. Descriptive statistics summarized the data. Logistic regression was used to model excess body weight resulting into adjusted odds ratios with their 95% confidence intervals and significant level was set at p-value<0.05.

**Results:**

the proportion of adolescents with excess body weight (BMI >+1SD) was 23%. The majority (63%), reported unhealthy dietary habits while half (51.5%) of them had moderate level of knowledge on healthy eating. Compared to working as a civil servant, the odds of having excess body weight among girls whose mothers/female guardians were housewives was less by 60% (aOR=0.4, 95%CI: 0.2, 0.9). Furthermore, the odds of having excess body weight among adolescents eating unhealthy foods were almost six times higher compared to their peers on healthy diet (aOR=5.8, 95%CI: 2.9, 11.3).

**Conclusion:**

prevalence of excess body weight among adolescent girls in northern Tanzania is high. Unhealthy dietary habits and mother's/female guardian's occupation were significant correlates of excess body weight. We recommend platforms to inform adolescents on the importance of proper food intake and to advance knowledge on dangers of excessive weight gain as a strategy towards prevention of nutrition-related diseases.

## Introduction

Excess body weight (EBW) that encompasses overweight and obesity, is recognized as one of the risk factors for various non-communicable diseases (NCDs) such as type 2 diabetes mellitus, osteoarthritis, cardiovascular-related diseases and certain types of cancers. These conditions lead to premature death as well as decrease in quality of life. Traditionally, an increased prevalence of overweight and obesity was initially only detected in adults. However, there has been a tremendous increase in the prevalence among adolescents worldwide. Thus, EBW is currently among issues of public health concern in adolescents, especially in low- and middle-income countries [1].

Demographically, an adolescent is an individual aged between 10 to 19 years. According to World Health Organization (WHO), there are about 1.8 billion adolescents, which is approximately 20% of the world´s total population. Out of these, 90% are from low and middle income countries [2]; and in Tanzania, adolescents constitute about 20% of the country´s population [3]. Adolescence is a period of transition from childhood to adulthood during which they undergo intense growth with increased nutrient demand. It is during this period when adolescents gain about 15% of their ultimate adult height and 50% of their weight [4]. Changes acquired during adolescence can lead to serious non-communicable diseases (NCDs) in late stages of life. The effects of inappropriate body weight among adolescent girls can persist throughout their later reproductive life and to their future generations [5].

The prevalence of EBW in children and adolescents is increasing, entailing health risks [6]. Low- and middle-income countries are experiencing a dramatic increase as a result of nutrition transition. This transition is characterized by a trend towards consumption of a diet high in fat, sugar and refined foods and low in fiber and declining level of physical activity [1,7]. There is clear evidence indicating increased likelihood of future generation childhood and adolescents´ obesity. In Tanzania, about 20% of non-pregnant adolescent girls aged 15-19 years residing in urban areas have excess body weight [8]. In the present society, where so much importance is given on appearance and looks, overweight and obese individuals often experience discrimination and rejection at school as well as at their workplaces [9].

Adolescence period is a critical window of opportunity for inculcating healthy lifestyle and behavioral changes since at this time, their cognitive as well behavioral domains can best adopt necessary and healthy habits in the long term. Indoctrinating such healthy behaviors and practices during early phases of life is the most impacting and cost-effective way to deal with the forthcoming problems [10]. Nutrition knowledge may be one of the catalysts towards an individual´s dietary habits [11].

Addressing the nutrition status of adolescents, specifically of girls, may contribute to breaking the vicious circle of intergenerational malnutrition, poverty and chronic diseases. If adolescents are well nourished, they can make optimal use of their skills, talents and energies. Promoting healthy lifestyle behaviors, along with healthy dietary choices, could modify obesity risk and improve the health of future generations [12]. This study hence assessed the magnitude and correlates of EBW among secondary school adolescent girls in order to inform stakeholders developing strategies for mitigating overweight and obesity among this population sub-group.

## Methods

**Study design and study setting:** a school-based cross sectional analytical study involving secondary school adolescent girls was conducted in Moshi municipality in northern Tanzania. The age-range of the respondents was within the World Health Organization (WHO) age category for adolescents, 10-19 years. Moshi Municipality is an urban district and is among the seven districts of Kilimanjaro region. The Municipality has a total of 43,205 adolescents. It has 30 secondary schools, of which 18 are boarding schools. However, the study focused only on girls in day-schools, since unlike their counterparts in boarding schools, they are more exposed to different types of foods and a variety of choices.

**Sample size estimation and sampling procedure:** we estimated a sample size of 427 adolescent girls based on the formula Z^2^p(1-p)/d^2^ where Z is a critical value of the normal distribution at 5% level of significance; p is estimated percent of overweight/obese adolescents, 20.4% [8]; and 4% for d, an estimated margin of error. We obtained a minimum sample size of 385. However, in order to account for unforeseen non-response of 10%, we adjusted the sample size to 428 respondents.

We used stratified and three-stage cluster sampling methods to select the adolescent girls. In the first step, we stratified the available 12-day schools into public (eight) and private (four). Half (50%) of schools in each of the two strata were selected randomly. With equal allocation, we aimed to have 72 girls from each school. In the second stage, for each of the selected secondary schools, we obtained the corresponding number of girls in each grade (form 1 to 4). We selected one grade at random. In the third stage, for each of the selected grade, we used simple random sampling to recruit respondents.

**Data collection:** we used a self-administered questionnaire to gather information from the adolescent girls in August 2019. This tool had structured questions organized into several sections including background characteristics, dietary habits, knowledge on healthy eating and anthropometric measurements.

Dietary habits are habitual decisions of adolescents regarding what foods they eat. These were measured by using selected food items from the Food Frequency Questionnaire (FFQ) adopted from the 2011 Food and Agriculture Organization Guideline [13]. These comprised of five food items; namely, fried meat, French fries, soda, vegetables and fruits. For each item, the participant was asked to recall the frequency of consumption within one week preceding the survey. On one hand, healthy dietary constituted of the consumption of fruits and vegetables and breakfast more than three times in the preceding week and consumption of fried foods and soft drinks less than three times during the same period. On the other hand, unhealthy dietary habits comprised of an eating pattern which involved regular consumption of French fries, fried meat and sodas; coupled with eating fruits and vegetables three times or less in the preceding week.

In this study, knowledge on healthy eating implied possession of appropriate information or understanding to enable healthy food choices, including food diversity and high intake of fruits and vegetables. We assessed this knowledge based on previously validated questions on nutrition for adolescents [14]. The assessment used nine items consisting of complete phrases of correct statements as follows: eating breakfast can improve school performance; fast foods like French fries are not suitable for balanced nutrition; it is recommended to drink about 8-10 glasses of water every day; amount of salt to be taken in a day should not exceed 6 grams; there are five major groups of foods; overweight and obese people have more health problems than normal weight people; eating vegetables and fruits can reduce occurrence of non-communicable diseases such as cancers; high sugars, fats and salt are associated with health problems; and five portions of fruits and vegetables per day are recommended for good nutrition. We instructed the adolescents to answer ‘yes´, ‘no´ or ‘do not know´ for each item. Anthropometric measurements were taken using the UNICEF/WHO approved electronic weighing scale and height board following standard procedures. Respondents were weighed while in bare-foot and with light clothes. Measurements were recorded to the nearest 0.1kg. We took the height of each respondent using a Harpenden Stadiometer (Holtain Ltd-UK), which was placed firmly against a wall and the results were recorded to the nearest 0.1cm.

**Data processing and analysis:** we processed and analyzed part of the data using Statistical Package for Social Sciences (SPSS) computer software (Version 24). Frequencies were generated for background information, knowledge on healthy eating and dietary habits. We created a composite variable to classify food items eaten by each adolescent into healthy and unhealthy foods. Level of knowledge on healthy eating was computed from the nine questions, whereby the maximum score was 9 for the entire scale. Each individual´s total score was grouped as 0-3, 4-6 and 7-9 representing low, moderate and high levels of knowledge respectively. Body mass index (BMI) was calculated as weight in kilograms divided by the square of height in meters. In computing BMI for age, the WHO AnthroPlus calculator was used with the recommended WHO reference +1SD cut off for 5-19 years. This is equivalent to the 85^th^ percentile and it coincides at 19 years with the adults´ cut off of BMI=25kg/m^2^, which is the cut-off for EBW.

We presented categorical variables using frequency tables and examined differences between proportions using Chi-square test. Independent correlates for EBW among the respondents were determined using bivariate logistic regression in the multivariable model. Factors with p-value <0.2 in the bivariate analysis were selected to enter into the regression model. However, additional factors that have been documented in literature to influence weight gain such as the type of school as well as education and occupation of the father were also included in the multivariable model. Adjusted odds ratios and their corresponding 95% confidence intervals (CI) are presented as both measures of association and their strengths. The significance level was set at 5%.

**Ethical approval:** Muhimbili University of Health and Allied Sciences (MUHAS) Institutional Review Board granted ethical clearance (Ref: DA.287/298/01A). Permission to conduct the study was requested from all relevant government authorities in Kilimanjaro region, Moshi Municipality and school administration. Prior to data collection, each of the selected girls was informed of the importance of the study and was requested to inform her parents/guardians on the same and this encouraged them to sign the consent form. Anthropometric measurements were taken while ensuring privacy. Each adolescent was assured of confidentiality during and after data collection.

## Results

**Background characteristics of study participants:** the study included 400 adolescent girls (participation rate=93.5%). Of these, 303 (75.3%) were from public schools. Table 1 summarizes the background characteristics of the study participants. Their ages ranged between 13 and 19 with mean, 15.1 ± 1.5 years. More than half of the girls, 239 (59.8%) and 233 (58.3%) reported their fathers or male guardians and mothers or female guardians respectively having at least secondary education. Most adolescents reported that their mothers/female guardians were involved in business 255 (63.8%), whereas almost a third of the fathers/male guardians were involved in either the private sector 127 (31.8%) or business 126 (31.5%).

**Table 1:** distribution of respondents by their background characteristics (N=400)

Characteristic	Number (%)
**Age group (years)**	
<15	97 (24.3)
15-19	303 (75.7)
**Grade**	
Form one	90 (22.5)
Form two	121 (30.3)
Form three	95 (23.8)
Form four	94 (23.5)
**School type**	
Public	301 (75.3)
Private	99 (24.7)
**Father/male guardian education**	
Secondary and above	239 (59.8)
Primary and below	161(40.2)
**Mother/female guardian education**	
Secondary and above	233 (58.3)
Primary and below	167 (41.7)
**Father/male guardian occupation**	
Civil servant	53 (13.3)
Private sector	127 (31.8)
Farmer	43 (10.8)
Business	126 (31.5)
Don't know	51 (12.8)
**Mother/female guardian occupation**	
Civil servant	39 (9.8)
Private sector	44 (11.0)
Farmer	29 (7.3)
Business	255 (63.8)
Housewife	18 (4.5)
Don't know	15 (3.8)

**Magnitude of overweight and obesity:** of the 400 respondents in this study, 92 (23%) (95%CI: 19.0, 27.5) had EBW relative to the standard normal distribution of WHO reference population for age of children aged 5-19 years. In Table 2 we present results of bivariate analysis of the relationship between EBW and selected background variables. The proportion of girls with EBW in the 15-19 age group was slightly lower than among the younger adolescents (21.1% vs. 28.8%; p=0.12). Compared to working in the other sectors, higher proportions of girls with EBW were observed among respondents whose fathers/male guardians or mothers/female guardians worked as civil servants, 16 (30.2%) and 16 (41.0%) respectively. However, only mother´s/female guardian´s occupation was significantly associated with adolescent´s EBW (p<0.03). Similarly, compared to girls whose dietary habits were classified as healthy, 13 (8.8%), the proportion of respondents with unhealthy dietary habits, 79 (31.3%) was significantly higher (p<0.001).

**Table 2:** association between excess body weight and selected characteristics (N=92)

Characteristic	Excess body weight, n (%)	p-value
Age group (years)		0.12
<15	28 (28.9)	
15-19	64 (21.1)	
School type		0.95
Public	69 (22.9)	
Private	23 (23.2)	
Father/male guardian education		0.33
Secondary and above	59 (24.7)	
Primary and below	33 (20.5)	
Mother/female guardian education		0.07
Secondary and above	61 (26.2)	
Primary and below	31 (18.6)	
Father/male guardian occupation*		0.32
Civil servant	16 (30.2)	
Private sector	31 (24.4)	
Farmer	8 (18.6)	
Business	30 (23.8)	
Mother/female guardian occupation		0.03
Civil servant	16 (41.0)	
Private sector	10 (22.7)	
Farmer	6 (20.7)	
Business	55 (21.6)	
Housewife	5 (27.8)	
Dietary habits		<0.001
Healthy	13 (8.8)	
Unhealthy	79 (31.3)	
Knowledge on healthy diet		0.13
Moderate	41 (19.8)	
High	51 (26.3)	

* 7 respondents did not know the occupation of the male parent/guardian

**Dietary habits among the study participants:** over three-quarters 307 (76.8%) of the girls reported a steady breakfast before going to school whereas slightly above half of them 217 (54.2%) reported consuming French fries regularly. A further 168 (42.0%) of the adolescents reported to frequently drink soda, four or more times a week. More than half of adolescents, 252 (63.0%), were categorized as having consumed unhealthy diets within the week preceding the study.

**Knowledge on healthy diet:** overall, about half of the participants 206 (51.5%) had moderate level of knowledge on healthy eating, while the remaining 194 (48.5%) were classified as having high level of knowledge on healthy eating. Regarding knowledge on the individual knowledge-scale items (Table 3), majority of the adolescents knew the importance of eating breakfast before school, 395 (98.8%). Furthermore, 371 (92.8%) knew that food items with high sugars, fats and salt were associated with health problems. Slightly more than half of the girls 211 (52.8%), were aware of the untoward health effects of eating fast foods such as French fries and drinking soda; and that overweight and obese people have more health problems compared to persons with normal body weight 269 (67.3%).

**Table 3:** proportion of respondents with correct scores on healthy eating questions (N=400)

Item	Number (%)
Eating breakfast can improve school performance	395 (98.8)
Fast foods consumption for example French fries not suitable for balanced nutrition	211 (52.8)
It is recommended to consume about 8-10 glasses of water everyday	337 (84.3)
Amount of salt to be consumed in a day should not exceed 6gm	309 (77.3)
There are 5 major groups of food	294 (73.5)
Obese and overweight people have more health problems than normal weight people	269 (67.3)
Eating vegetables and fruits can reduce the risk of non-communicable diseases such as cancer	273 (68.3)
High sugars, fats and salt associated with health problems	371 (92.8)
Around 5 portions of fruit and vegetable consumption are recommended for good nutrition	255 (63.8)

**Correlates of excess body weight:** in Table 4, we present results from univariate and multivariable logistic regression analyses with EBW being the dependent variable. After adjusting for other variables, significant correlates of EBW were occupation of the mother/female guardian and dietary habits. Compared to working as a civil servant, the odds of having EBW among girls whose mothers/female guardians had other occupations were lower by about 60-70%. However, the only significant association with EBW was among adolescents whose mothers/female guardians were housewives (aOR=0.4, 95%CI: 0.2, 0.9). Additionally, the odds of having EBW among adolescents classified as eating unhealthy foods were almost six times higher compared to their peers whose diets were categorized as healthy (aOR=5.8, 95%CI: 2.9, 11.3). Even though the level of knowledge on health eating was not a significant correlate of EBW, nevertheless, girls with high knowledge had almost two times higher odds of having EBW (aOR 1.6, 95%CI: 0.9, 2.7).

**Table 4:** logistic regression of correlates of excess body weight

Characteristic	Crude OR (95% CI)	Adjusted OR (95% CI)
**Age group (years)**		
<15	Reference	Reference
15-19	0.7 (0.4, 1.1)	0.7 (0.4, 1.4)
**School type**		
Public	Reference	Reference
Private	1.0 (0.6, 1.7)	1.1 (0.6, 2.0)
**Father/male guardian education**		
Primary and below	Reference	Reference
Secondary and above	0.8 (0.5, 1.3)	1.2 (0.6, 2.3)
**Mother/female guardian education**		
Primary and below	Reference	Reference
Secondary and above	0.6 (0.4, 1.0)	0.7 (0.4, 1.4)
**Father/male guardian occupation**		
Civil servant	Reference	Reference
Private sector	0.7 (0.4, 1.5)	1.2 (0.5, 2.8)
Farmer	0.5 (0.2, 1.4)	0.8 (0.2, 2.7)
Business	0.7 (0.4, 1.5)	1.0 (0.4, 2.4)
**Mother/female guardian occupation**		
Civil servant	Reference	Reference
Private sector	0.5 (0.2, 1.3)	0.3 (0.1, 1.1)
Farmer	0.4 (0.1, 1.1)	0.4 (0.1, 1.8)
Business	0.6 (0.2, 1.9)	0.3 (0.1, 1.4)
Housewife	0.4 (0.2, 0.8)	0.4 (0.2, 0.9)
**Dietary habits**		
Healthy	Reference	Reference
Unhealthy	4.7 (2.5, 8.9)	5.8 (2.9, 11.3)
**Level of knowledge on healthy diet**		
Moderate	Reference	Reference
High	1.4 (0.9, 2.3)	1.6 (0.9, 2.7)

## Discussion

The aim of the study was to determine the magnitude of excess body weight (EBW) and associated factors among secondary school adolescent girls in an urban setting in northern Tanzania. The findings show that 23% of the girls have EBW. Significant correlates of EBW are occupation of the mother/female guardian and the individual dietary habits. The high prevalence of EBW among the girls in the current study is of concern since overweight and obesity in adolescence are likely to continue into adulthood [15]. Available literature indicates that the proportions of adolescents with EBW has increased substantially in developed countries such that in 2013, about 23% of boys and girls were overweight or obese [6]. The proportion of adolescents with EBW in our study is slightly higher than the 14% and 17% documented rates from some other areas of Tanzania [16,17]. The difference could be due to study settings and availability of fast foods as the current study was purely in an urban setting. However, similar high proportions of school children with EBW have been reported in other areas of Tanzania and Kenya [18,19], suggesting that the problem is wide spread.

Children and adolescents in developing countries are prone to sugary, high fat, energy-rich and micronutrient-poor foods that are less costly and lower in nutrient quality. These dietary habits in combination with other factors result in substantial upsurge of overweight/obesity. Most chronic diseases in adulthood originate from dietary practices which are mainly formed during childhood. In our study, a high proportion of the adolescents reported unhealthy dietary habits, constituting of occasional to regular consumption of fast foods and sweetened beverages. Likewise, high intake of fried foods and soft drinks, as well as less consumption of fruits and vegetables have also been reported among adolescents in other parts of Tanzania, Kenya and India [7,17,20,21]. These types of unhealthy eating behaviors could be a result of poor perception of the consequences of overweight and obesity as well as the nutrition transition whereby fast foods are easily available, especially in the semi-urban and urban areas.

In this study, we observed a moderate level of knowledge on healthy eating among the adolescent girls. Similar results have been reported by previous researchers in other areas [20,22]. Lack of adequate nutrition knowledge has implications on dietary habits. Surprisingly, in the current study, the odds of having EBW among girls who had high level of knowledge on healthy eating were almost two times higher (aOR=1.6, 95%CI: 0.9, 2.7) compared to colleagues with moderate knowledge. This suggests that the girls are not translating their nutritional knowledge into appropriate practice. The current study has shown no significant association between age and prevalence of EBW, contrary to observations in Mwanza, Tanzania [16] where older adolescents aged between 18-19 years were significantly more overweight/obese compared to younger adolescents, 16-17 years. This difference could be attributed to the type of respondents, as the Mwanza study involved males and females from both boarding and day schools whereas the current study focused on only adolescent girls in day schools.

Compared to learning in public secondary schools, learning in private schools has been found to be associated with a three-fold increased risk of EBW among adolescents in Ethiopia [23,24]. Likewise, findings from studies in Dar es Salaam and Dodoma, in Tanzania revealed increased risk of overweight and obesity in private school students compared to their peers in public schools [25,26]. However, similar to a study in Babati [17], the current survey shows no association between EBW and school type. This may be due to differences in socio-economic status between families of these adolescents as well as availability of fast foods especially in the large cities. In our study, parental level of education is not significantly associated with EBW among the adolescent girls. These findings are similar to observation in Dar es Salaam [25] but different from Babati [17] and Ethiopia [24]. The later studies documented a significant risk of overweight/obesity among adolescents whose parents had higher education compared to those whose parents were less educated or illiterate. Adolescent girls whose mothers/female guardians are housewives have lower odds of having EBW compared to girls whose mothers/female guardians work in the public sector or are involved in any other occupation. Likewise, a related survey in Kenya among school children showed that unemployed mothers had significantly higher proportions of children with normal weight [18]. This suggests that socioeconomic status of the mother is associated with EBW in children and adolescents.

Unhealthy dietary habits among adolescent girls in this study are significantly associated with EBW. Girls who were classified as consuming unhealthy diets have almost six odds of having EBW (aOR=5.8, CI 2.9, 11.3) compared to their counterparts. Higher odds of overweight and obesity among adolescents consuming unhealthy diets have also been documented by allied studies in Zanzibar, India and Ethiopia [7,21,24]. Adolescents require a balanced intake of protein, fat and carbohydrates whereas over-consumption of one or more macronutrients may contribute to weight gain. However, knowledge on healthy diet in this study is not translated into healthy eating, which is comparable to observations elsewhere [20].

This study has some potential limitations. One, we are unable to completely rule out recall bias when collecting data on type and frequencies of foods in a period of seven days. Such information depended on the adolescent´s ability to recall on past events. Second, although we strictly followed standard procedures in taking anthropometric measurements, including calibrating the weighing scale regularly, still intrinsic measurement errors may have affected the study results.

## Conclusion

This study revealed that prevalence of excess body weight among adolescent secondary school girls in an urban setting in northern Tanzania is high. Unhealthy dietary habits; and mother/female guardian´s occupation are significant correlates of EBW. Promoting healthy dietary habits may be an effective intervention. We therefore, recommend platforms to inform adolescents on the importance of proper food intake and to advance knowledge on dangers of excessive weight gain as a strategy towards prevention of nutrition-related diseases.

### What is known about this topic

- Childhood and adolescent overweight and obesity are becoming a global public health problem;
- Previous studies have shown that adolescents lack adequate knowledge on healthy eating.

### What this study adds

- High proportions (23%) of secondary school adolescent girls in northern Tanzania have excess body weight;
- Knowledge on healthy eating habits among adolescent girls, as most studies have mainly focused only on establishing the prevalence of EBW;
- The influence of mother´s/female guardian´s occupation on the likelihood of an adolescent becoming overweight or obese.
